# Pregnancy from a Legal Standpoint

**Published:** 1909-02-13

**Authors:** 


### Medico-Legal Points.

### PREGNANCY FROM A LEGAL STANDPOINT.

Consent to Medical Examination.

Medical men who are called upon to make examinations of living persons in connection with the organs of generation should bear in mind that they must in all cases obtain the person's express consent, and in the case of children under tutelage the express consent of parent or guardian, without which they render themselves liable to prosecution for indecent assault, even when acting under the order of a magistrate or judge. In *Agnew* v. *Jobson* (1877, 13 Cox's Crim. Cas. 625) it was held that a magistrate has no right to order an examination of the person of a prisoner. An examination by medical men, in pursuance of such an order, of the person of a female in custody, upon the charge of concealing the birth of her illegitimate child, constitutes an assault.

In a civil action the prisoner recovered damages against doctor, police, and magistrate. In the case of an unconvicted prisoner, or of a person merely suspected of being implicated in a charge, it is absolutely *ultra vires* for a policeman, coroner, coroner's officer, magistrate, or judge to make an order for the personal examination of anyone. Consent should, and must, be obtained. Such consent should be given in writing preferably, or in the presence of disinterested witnesses in any case; and, if the case is in the slightest degree doubtful from any cause whatever, the medical man must insist upon its being given in writing, and upon a guarantee being given against any proceedings. Such consent must not be obtained under any undue moral pressure or duress; it must be freely given after a full explanation of the circumstances for which it is asked and of the consequences that may result from it.

Police Orders.

The following is from the Police Orders issued to the Metropolitan Police as to the medical examination of prisoners:—

"The law officers of the Crown having advised the Secretary of State that it is expedient that a medical examination of prisoners charged with such offences as rape should be made, police inspectors must see that such examination is made in such cases where a prisoner consents. With regard to the offences to which this order is applicable it is impossible to give a complete list, but it includes unnatural offences and rape, and all offences under the Criminal Law Amendment Act, 1885, and all cases in which the examination under this order, without the prisoner's affirmative consent, seems likely to furnish evidence as to the prisoner's guilt or innocence. If a prisoner consents to such examination he is to be told that if he desires the attendance of a qualified medical man on his behalf an opportunity for such attendance with the divisional surgeon will be given, and arrangements are to be made accordingly. An entry is to be made and signed by the inspector at the time of every proposal for a medical examination, and of the fact of consent or refusal being given by the prisoner in his presence; also of the offer made to the accused to allow a qualified medical man to attend on his behalf, and of the fact of the accused having accepted or rejected such offer, and such entry shall be read to the accused person. If an examination is made and a committal for trial takes place the officer must attend the trial, and have the entry with him to prove the consent. The divisional surgeon must make a separate entry in his private memorandum book of the result of any examination, and he must be informed of the time and place when his attendance will be required to give evidence before the magistrate. By an examination carefully conducted under these rules, an innocent man cannot suffer, and such examination would often furnish cogent evidence against the guilty. This order does not interfere with the accustomed police or other search of prisoners charged with felony with a view to discover evidence bearing on the charge, under paragraph 36 of Police Order 'Prisoners,' which is not applicable to a medical examination; nor does this order interfere with the accustomed practice as to medical aid. Where a prisoner is in custody on any charge in which a personal medical examination may be material to the accused, but not being an offence to which paragraph 2 of this order extends, if the accused or their friends (acting with their consent) expressly desire such examination, it is to be made either by the divisional surgeon or medical man attending on the part of the accused, in which latter case the divisional surgeon is also to be present, and the officer in charge of the station is expressly to enter in the Occurrence Book the request of the accused and the compliance with it, and to report the facts."

Comments by an Expert.

On this Sir Thomas Stevenson commented as follows:—

"All these minute directions, so far as they apply to London divisional surgeons, apply with equal force to every surgeon who is asked to examine a male prisoner. This consent is all the more important because many men, especially young men, finding themselves in custody, might submit to an examination under terror, which is not consent. It is not only necessary to obtain the consent of any prisoner to an examination, but it is very desirable to caution him that the examination may be evidence in his favour or against him, and that in either case the surgeon will be bound to tell the truth. In a case of unnatural offence tried by Hawkins, J., at the Central Criminal Court in 1890 a divisional surgeon of police was severely censured by the judge for not cautioning the prisoner as to the result of the examination, and so taking advantage of the prisoner's ignorance. Men who commit these crimes frequently are well aware of the importance of an examination as *prima facie* evidence in their favour should it be negative, and it is surprising how often a criminal assault has been committed without leaving any trace upon the accused. But refusal to submit to an examination is not necessarily an admission of guilt. For instance, a prisoner may be suffering from venereal disease, and be unwilling that this should be disclosed, and yet may be innocent of the crime of which he is charged. If the complainant has venereal disease too this coincidence might be false as well as true evidence. There is not unanimity of opinion among the English judges as to necessity of obtaining consent before examining a prisoner, and at a trial in 1890 for murder following rape Huddleston, B., laid down that the police had as good a right to examine the prisoner's person as his clothes. But most judges are of a diverse opinion, and under such circumstances medical practitioners would act wisely in being on the safe side."

Apparently the only case in which a person is compelled to submit to a medical examination is that of a female sentenced to death who pleads pregnancy. In such a case a jury of matrons is empanelled, and from their own observation and the medical evidence return their verdict.

### Jury of Matrons.

In two cases—the one criminal and the other civil—the question whether a given woman is with child or not is submitted to the discussion of a jury of matrons or other "discreet women." In the first case, the use of this means of inquiry is obligatory, and by the Court, in the second, optional, and upon the petition of a private litigant. The procedure is of high antiquity, stated by Blackstone (4 Com. 395) to have prevailed, in pleas of the Crown, "as early as the first memorials of our laws will reach," and by Sir Lloyd Kenyon declared to be, as to its employment in civil suits, "coeval with the common law" itself. Its origin is either to be found in the analogy suggested by the ordinary trial by jury, and in the idea that a person should be tried by his (or her) peers, or with more probability in the practical reason that, in the matter to be decided, matrons were regarded, however mistakenly, as experts. In early ages, when medical knowledge was rare in amount and poor in quality, resort to a jury of matrons was, no doubt, the best and surest means of inquiry. Modern experience, however, does not justify the continued existence of a procedure which has often led to wrong results, and for which the medical profession furnishes a more easily available, as well as a more reliable, substitute.

In every capital case where the culprit is a woman upon conviction, and after sentence of death has been pronounced, the clerk of the Court is to ask the prisoner, "What have you to say for yourself in stay of the execution of the sentence which has been given against you?" It is in answer to this question that the prisoner is to plead pregnancy if she chooses to do so. When a plea of pregnancy has been thus raised *ad retardationem executionis*, the clerk is to empanel forthwith from the bystanders present in Court (*de circumstantibus*) a jury of twelve matrons or "discreet women" (2 Hale, P. C. 413).

The course usually followed is exemplified in *Wycherley's Case* (1838, 8 C. and P. 262): there Gurney, B., said, "Let all the doors be shut, and no one be suffered to leave the Court," that is to say, to prevent the ladies present as sightseers from withdrawing, and so declining an unwelcome office. Too often an unseemly scene follows. The Annual Register for 1872 (pp. 3, 4, and p. 200 of the Chronicle, in the same volume), describing the trial of Christina Edmonds and her plea of pregnancy, says that "after about twenty minutes a dozen well-to-do and respectably dressed women were captured and directed to enter the jury-box." The jury being now empanelled, the forewoman is first sworn separately and in some detail, and then the other jurywomen more shortly, to "search and try the prisoner at the bar whether she be with child of a quick child or no" (Archbold, 1905, 23rd ed. p. 229). A bailiff is then sworn to keep the jury "without meat," etc., according to the usual form with one exception; for the other runs, "You shall not suffer any person but the prisoner to speak to them." The jury withdraw with the prisoner to make their examination. But they may desire, and will be accorded, the testimony of a qualified medical man, and probably in these days such testimony would in practice be considered indispensable. In *Webster's Case* (C.C.C. Sess. Pap. vol. xc.) the verdict of the jury was given upon their own examination and upon the evidence of Thyrza Belsham, one of the wardresses of Newgate, and Mr. Bond, the surgeon, who examined the prisoner. Were this not so, grave mistakes would occur, such as in the past have occurred; for "perhaps there is no subject on which average women display greater ignorance than on questions connected with pregnancy" (Tidy's Leg. Med. 1883, Part. II. p. 107). The books make mention of an early case where a mistake was made in this matter at Aylesbury (1 Hale P. C., p. 369). And in modern times mistakes have been made, *e.g.* in the *Norwich Case* (*R.* v. *Wright*, 1832, Med. Gaz., vol. xii., p. 22; Med. Times and Gaz., Jan. 27, 1872, p. 98), in which a jury of matrons wrongly found a prisoner not with child. So in *Hunt's Case* in 1847, where a negative verdict was returned, the woman had actually at the time passed the period at which "quickening" usually takes place (Taylor's Med. Jur., 4th edit., vol. ii. p. 161). The woman would have been executed but for the interference of the Secretary of State.

(*To be continued.*)

